# Factors Determining Suitable Landfill Sites for Energy Generation from Municipal Solid Waste: A Case Study of Jabodetabek Area, Indonesia

**DOI:** 10.1155/2022/9184786

**Published:** 2022-02-27

**Authors:** Muhammad Achirul Nanda, Arif Kurnia Wijayanto, Harry Imantho, Leopold Oscar Nelwan, I Wayan Budiastra, Kudang Boro Seminar

**Affiliations:** ^1^Department of Agricultural and Biosystem Engineering, Faculty of Agro-Industrial Technology, Universitas Padjadjaran, Jatinangor 45363, Indonesia; ^2^Environmental Research Center (PPLH), IPB University, Bogor 16680, Indonesia; ^3^Remote Sensing & Ecology Laboratory, Southeast Asian Regional Centre for Tropical Biology, Bogor 16134, Indonesia; ^4^Department of Mechanical and Biosystem Engineering, Faculty of Agricultural Engineering and Technology, IPB University, Bogor 16680, Indonesia

## Abstract

Most municipal solid waste (MSW) is found to be dominated by organic debris, which has excellent potential as an energy source. However, the main problems of this material are poor planning, urban expansion, and lack of management skills. All these problems are presently being encountered by the regional governments of Jakarta, Bogor, Depok, Tangerang, and Bekasi city (known locally as Jabodetabek), Indonesia. In the MSW management system, a vital planning protocol is reportedly assessing suitable landfill sites for energy generation, although this selection process is still a complex task that should consider various factors, such as environmental, social and safety, and economic variables. Therefore, this study aims to examine various factors in determining a suitable location for landfills. It also aims to identify the various factors required for MSW energy generation. Based on this study, a multicriteria decision analysis (MCDA) approach was applied to weigh the factors determining the appropriate location. This approach is popular in decision-making due to evaluating the complexity of multidimensionality factors. The results showed that 3 factors and 14 subfactors were formulated and structured in the MCDA hierarchy, with their information obtained to create pairwise comparisons by 10 involved experts. In this study, the MCDA output was the weight value associated with a systematic priority level, indicating that the environment was the highest factor in determining a suitable landfill site for energy generation. In addition, the weight factors were used for overlay analysis, in determining the suitable site for future energy generation studies.

## 1. Introduction

The municipal solid waste (MSW) is a set of numerous solid debris produced by cities, factories, and different types of household activities [1]. This material contains paper, glass, ferrous metals, aluminium, tin, copper, textiles, rubber, plastics, food, animals, and plants. It is also averagely dominated by organic waste (70%), compared to inorganic materials (30%). Subsequently, biogas, incineration, gasification, and composting are among the technologies used to convert MSW organic materials into energy sources [2]. These are often environmentally friendly, due to reducing greenhouse gas emissions from waste. According to Kumar and Ankaram [3], gasification technology produced 1,000 kWh of electricity per ton of MSW. However, an essential part of the government's significant concern, that is, the MSW management system, is being observed to convert waste into energy. This is because the effective management of MSW requires a good understanding of the quality and quantity of waste, economic cost, and the environmental impacts of treatment methods [4]. Most MSW problems in certain regions such as developing countries are severe and disorganized, due to poor planning, urban expansion, and lack of management skills. One of the planning protocols to complete the management system of this waste is the determination of a suitable landfill location, where various energy generation technologies should be installed. When a suitable location is found, the process of harvesting and distributing energy to the settlement is optimally, effectively, and efficiently operated. The selection of a suitable site requires an extensive evaluation process, where the location should consider various factors, such as the ecological and environmental, economic and infrastructural, social, natural disaster vulnerability, and biological conditions [5]. For example, the suitable landfill distances from a specific settlement and a river were >3,000 and 2,500 m, respectively, based on the ecological and environmental factors [6]. According to Wang et al. [7], social and economic factors also played an essential role in site selection, due to the financial aspects, costs, and conflicts. Therefore, the selection of a suitable site involves a reliable approach, which utilizes spatial, geographical, climatic, temporal, and attributive data.

Several scientific documents were widely reported based on the selection of a suitable site for landfills and also a ranking analysis of various factors. The case studies of these reports subsequently covered several locations in various regions, such as Antalya [8], Gondar town [9], Srinagar city [10], and Kupang [11] in Turkey, Ethiopia, India, and Indonesia, respectively. Therefore, this study aims to examine various factors in determining a suitable location for landfills. It also aims to identify the various factors required for MSW energy generation. Several previous studies were subsequently found to only examine specific locations, whose direct application to this present report was highly possible. Furthermore, the case study of this report is located in the densely populated areas of Indonesia, that is, Jakarta, Bogor, Depok, Tangerang, and Bekasi, with each location having a distinctive culture, characteristic, climate, lifestyle, and so on. This study subsequently claims novelty, that is, the factors identified to determine suitable landfill location for MSW energy generation, at Jakarta, Bogor, Depok, Tangerang, and Bekasi, Indonesia. To identify the priority factors in determining suitable energy generation sites, a multicriteria decision analysis (MCDA) is used, due to providing different options with a collection of weighting techniques. The application of this analysis also provides an advantageous method, which manages the assessment time and costs, minimizes errors, and improves decision-making accuracy. The strength of the MCDA is based on the ability to include both qualitative and quantitative criteria in the decision space. In this study, the analytical approach is instrumental in determining the appropriate landfill location, due to the occurrence of various inevitable factors such as environmental, social and safety, as well as economic conditions, which should be carefully considered and weighed. Therefore, this study aims to analyze various factors in the suitable landfill for MSW energy generation, using a multicriteria decision analysis approach. The weight value generated by each factor is also beneficial to the determination of a suitable site for energy generation.

## 2. Case Study

**Figure 1:**
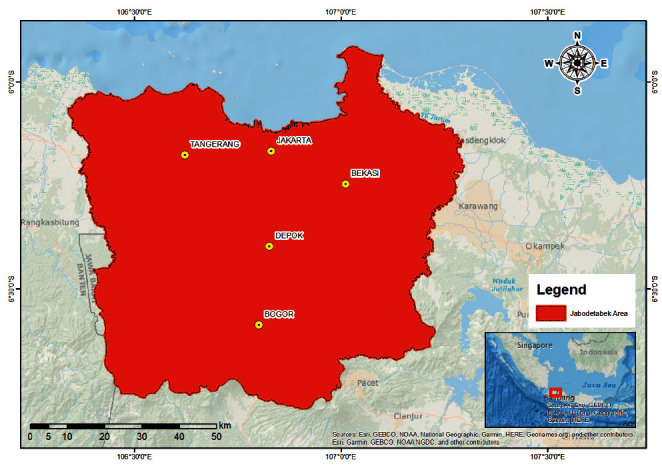
The study area.

**Figure 2:**
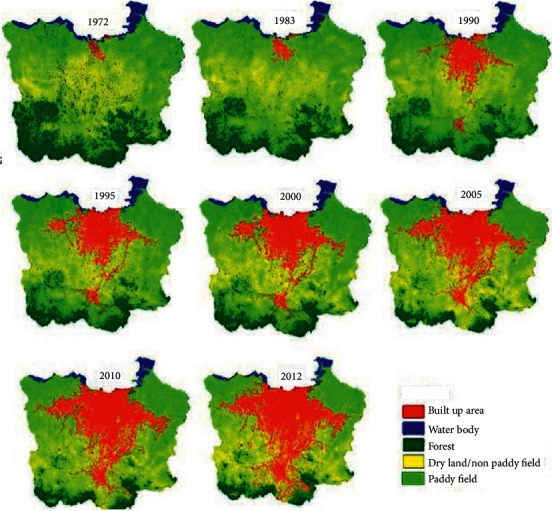
The map of land use/cover change in Jabodetabek in 1972–2012 (source: Rustiadi et al. [13]).

The case study is located at Jakarta, Bogor, Depok, Tangerang, and Bekasi city (known locally as Jabodetabek), Indonesia (Figure 1). In 2019, these locations covered a total area of 6,437.89 km^2^, with a population of approximately 34,564,239 [12]. Based on land use (Figure 2), Jabodetabek is reportedly undergoing significant changes, where many green areas or fertile agricultural lands are being converted into built-up regions (i.e., settlements, commercial services, buildings, industries, etc.). Since the expansion of urban areas, the land conversion for housing and built-up purposes has continuously increased between 1972 and 2012. Therefore, Jabodetabek is presently dubbed the most populous metropolitan area in Indonesia, due to playing critical roles in social, economic, and political aspects. However, the maintenance of this area and lack of planning capacity to deal with increasing complexity should be seriously considered. One of these maintenance problems is municipal waste management, which is still far from adequate.

Waste constitutes a significant problem in metropolitan cities, with vast solid quantities being generated in industries. This indicates that the annual waste generation in Jabodetabek is approximately 8.340 million tons/year, with the organic and inorganic constituents observed at 68 and 32%, respectively [14]. Based on the municipality, wastes are regularly gathered and buried in an unsanitary manner within an open area. This indicates that the entire Jabodetabek urban waste is collected at the Bantargebang landfill (around Bekasi city), with a daily volume of 7,500 tons/day. However, the Environment Agency (2021) stated that operations at the Bantargebang landfill should be stopped due to overcapacity, leading to the serious consideration of selecting an appropriate landfill site, to anticipate more complex problems. Another problem is that the landfill site is not specifically designed for energy generation installation, subsequently indicating that the studies related to environmental, social and safety, as well as economic factors are highly awaited by policymakers, in determining the suitable landfill for energy generation.

## 3. Methodology

**Figure 3:**
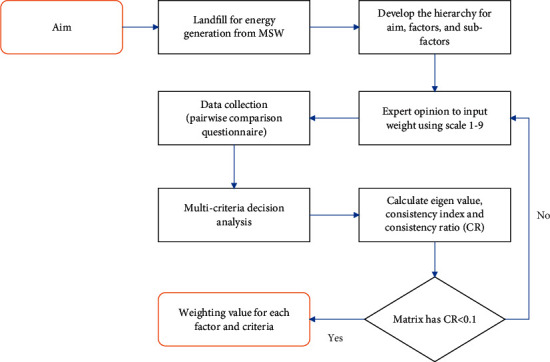
The flowchart to determine the factors of suitable landfill sites.

This study aims to identify the factors determining the location of suitable landfill sites for MSW energy generation in Jabodetabek, Indonesia. This study implemented a multicriteria decision analysis (MCDA) approach, whose explanation and procedures were gradually described. The visionary framework in siting the landfill for energy generation is shown in Figure 3, where the first step began with the hierarchical development of the aims, factors, and subfactors. This examines the various factors to determine the suitable location for a good landfill site for energy generation. By assigning an importance scale of 1–9, pairwise comparisons were conducted in this study by the involved experts. Furthermore, the whole comparative questionnaire was collected as the primary material in the subsequent analysis, that is, the MCDA approach. At this stage, a comparison matrix and acceptable level of consistency ratio (CR) were presented and calculated, respectively. This CR calculation was based on an evaluation of the consistency of the experts' judgment. When CR is less than or greater than 0.1, the judgments were found to be consistent or inconsistent, respectively. Based on inconsistencies, the experts were to reinput the weight on each factor. After the numerical analysis, the weight for each factor was produced and subsequently used as a reference to determine the landfill location. Figure 3 shows the total explanation of the performance stages.

### 3.1. Multicriteria Decision Analysis

The multicriteria decision analysis (MCDA) is a popular decision-making method for site suitability, due to the assessment of complex multidimensionality factors and criteria [7]. The weight of the factors and subfactors was determined to clarify the intensity of importance, where the analytical hierarchy process (AHP) was implemented to assess unequal essentiality [15]. In various domains such as the social, economic, agricultural, industrial, ecological, and biological systems, the AHP is widely used for practical MCDA methods [16–18]. It is also a descriptive decision analysis methodology, which calculates the ratio-scaled importance of alternatives, through pairwise comparison of factors and criteria [7]. The AHP is often combined with various systems to produce accurate decisions, such as artificial neural network [19], support vector machine [20–22], discriminant analysis [23], genetic algorithm [23], Gaussian process regression [24], and so on.

**Figure 4:**
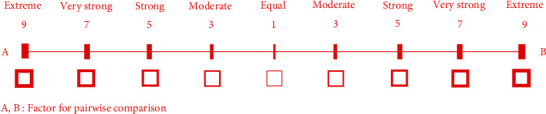
The scale of importance intensity for pairwise comparison.

Based on the AHP, the weight was determined by three stages. Firstly, the decision-making was broken into three levels, that is, aim, factors, and subfactors. This aim was to identify the indicators of selecting a suitable landfill site for energy generation, while factors and subfactors were the parameters used to achieve the aim. Secondly, the relative importance of the factor and subfactor was assigned. According to Wang et al. [7], the matrix of the pairwise comparisons (*D*) in AHP was expressed in equation (1), where *f*(*f*=1,2,3,…, *i*) was described as a factor (i.e., environmental, social and safety, as well as economic). Subsequently, the relative importance was scaled based on Figure 4. The pairwise comparison was also justified using an experts' importance scale 1–9, where the 1 indicated that one factor had equal importance to another. However, 9 showed the factor with extreme importance to others. This indicated that the higher scale value led to a greater importance level on the related factor.

(1) $$\begin{array}{c} D=\left[\begin{array}{cccc} \frac{f_{1}}{f_{1}} & \frac{f_{1}}{f_{2}} & \cdots & \frac{f_{1}}{f_{i}}\\ \frac{f_{2}}{f_{1}} & \frac{f_{2}}{f_{2}} & \cdots & \frac{f_{2}}{f_{i}}\\ \vdots & \vdots & \ddots & \begin{array}{c} \vdots \end{array}\\ \frac{f_{i}}{f_{1}} & \frac{f_{i}}{f_{2}} & \cdots & \frac{f_{i}}{f_{i}} \end{array}\right]. \end{array}$$

Based on this study, various experts were selected to determine the importance level of the factors, that is, waste care community, nongovernmental organization, department of agriculture, electrical industry, safety practitioner, construction professional and renewable energy scientist, and social, economic, and environmental factors. The total number of experts involved in this survey was 10, with each individual assigned to their respective field. These experts were comprehensively selected to represent the various diversity of each character, with most of them being dominated by the Jabodetabek metropolitan area. This was due to the location being the primary target for the study. Thirdly, the overall weights regarding the goal for each decision alternative were subsequently obtained. This indicated that the final weights of each factor and subfactor were between 0 and 1, with the sum of the weight = 1. The results showed that the factor and subfactor with the highest scores were the best alternatives. In this study, MCDA was performed using the Priority Estimation Tool (PriEst) software, which had easy built-in functions [25].

### 3.2. Determining Subfactors

**Figure 5:**
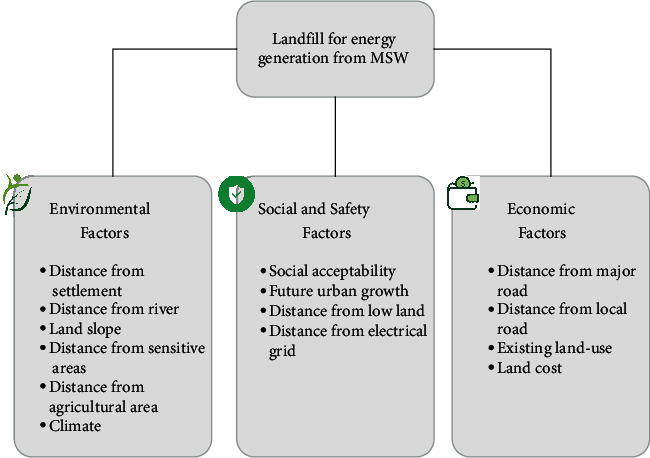
The factor hierarchy of site suitability for energy generation.

Although most present systems have good databases, they still lack the support of decision-making on the selection of suitable landfill sites for energy generation. This is due to site identification being a time-consuming process, which needs extensive data management. This shows that a comprehensive database should be able to provide information on the main factors of site selection. In this study, the subfactor structure was organized under three factors, namely, environmental, social and safety, and economic conditions. Based on Figure 5, the entire factors were defined and classified by the experts and literature. The compilation of the criteria for each factor is as follows: (i) the environmental factor contains the distances from the settlement, river, land slope, sensitive regions, an agricultural area, and climate; (ii) the social and safety factor contains public acceptability, future urban growth, as well as the distances from the low land and electrical grid; and (iii) the economic factor contains the distances from major and local roads, as well as the existing land use and cost. Therefore, a good landfill site should consider all the aforementioned factors and subfactors.

Based on this study, the environmental factor identified various societal aspects in the landfill site selection, idealistically indicating that the location should be far from the surface water bodies (lakes, ponds, rivers, etc.) and settlements, due to odour, nuisance, and public sentiment. The social and safety factor also identified various problems, for example, the future facilitation of power from the landfill location near the electricity grid to the community. Meanwhile, the economic factor was related to the financial restrictions and also included the consideration of present land use, as well as significant and local road distances. This indicated that landfills should be close to the road networks due to the construction and transportation costs [6]. The nomenclature for each factor and subfactor is shown in Table 1. This study contained three factors, namely, environmental, social and safety, as well as economic variables, whose subfactors were represented by “‘*V*,” “‘*S*,” and “‘*E*,” respectively. All these symbols were provided to ensure an easier understanding of the MCDA approach. In addition, the analysis was divided into three parts, that is, factor, subfactor, and combination categories.

### 3.3. Evaluation

The judgments executed by experts should be consistent, precise, and justifiable with narrow margin inconsistent values. At this stage, the MCDA introduced consistency ratio as an evaluation metric, to prevent subjectivity and inconsistency. The primary goal of this ratio is based on calculating the consistency level of the judgments, compared to the high samples of purely random decisions. This was calculated by dividing the CI (consistency index) and RI (random consistency index), as shown in the following equation:

(2) $$\begin{array}{c} \mathrm{CR=}\frac{\mathrm{CI}}{\text{RI}},\text{where }\mathrm{CI}=\frac{\lambda_{\max}}{n-1}, \end{array}$$

where *λ*_max_ and *n* represent the eigenvalue and matrix dimension, respectively. The results were considered inconsistent or consistent when the value of CR is greater than or less than 0.1, respectively. In addition, these experts were instructed to reevaluate the comparison matrices when inconsistencies are detected.

## 4. Results and Discussion

### 4.1. Expert-Based Survey

**Figure 6:**
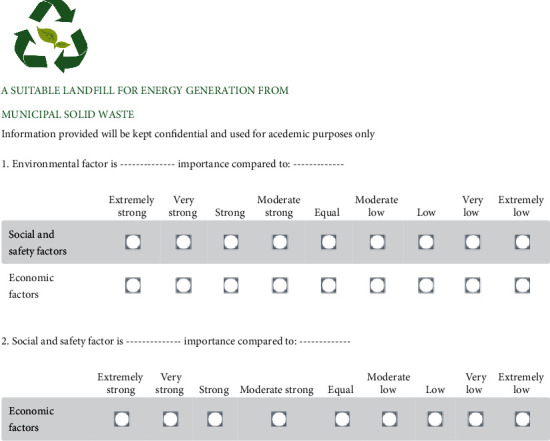
Part of the questionnaire in the MCDA.

Based on the MCDA approach, the collection of the questionnaire data was remotely and digitally carried out, with various factors and subfactors being compiled through web-based software, namely SurveyMonkey (https://www.surveymonkey.com). This ensured easier performances in efficiently tabulating, processing, editing, and monitoring data. This indicated that SurveyMonkey was a cost-effective and time-saving option for small assessment projects [26]. In this study, the complete document of the questionnaire was freely downloaded for other academic purposes at ResearchGate (https://bit.ly/AHPe-Questionnaire).  Figure 6 shows the part of the questionnaire where each expert had to answer. This was provided with the instruction to compare two information fields each time, regarding the target. To simplify the appearance, a convertible verbal judgment was utilized, due to the possibility of being transformed into future quantitative scale points. In addition, a justification copy was provided to the experts, which subsequently assisted them in producing consistent answers to pairwise comparison questions.

Based on Figure 6, all the justification points on the questionnaire were averaged and used as input for pairwise comparison. These were automatically performed using the easy built-in function in the SurveyMonkey software. In this study, each point in the pairwise comparison was retrieved, to produce the weights of each factor and subfactor through the open-source software of PriEst.

### 4.2. Pairwise Comparison

**Figure 7:**
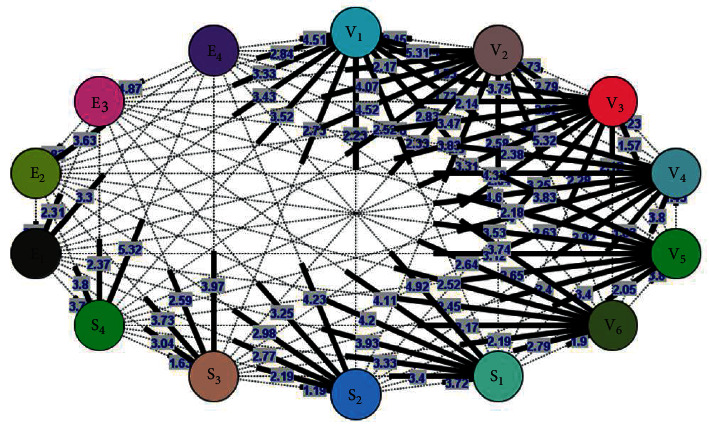
The score points as a representation of the importance level between the subfactors, to produce weights (imported from Table 6).

According to the MCDA approach, the experts were used to justify the importance level of each factor and subfactor, where a total of 273 pairwise comparisons were performed by 10 issue-related professionals. Based on Tables 2–6, a total of 9, 68, and 196 pairwise comparisons were comprehensively observed for the factor, subfactor, and combination categories, respectively. The points in each pairwise comparison were also the mean values calculated by the involved experts. For example, the mean of environmental factor comparisons with social and safety factors was 4.6 (Table 2). This indicated that the environmental factor was highly important than the social and safety variable. This was however not a problem when several pairwise comparison score points were not accessible on the verbal judgment lists. These points were then used in the AHP analysis to derive the weights. For example, Figure 7 visualized each point regarding the importance level, where the subfactors were compared to other variables.

The pairwise comparison was performed by comparing and determining the importance of all the factors. This indicated that the specification of the factors above and below 1 corresponded to higher and lower importance, respectively. According to Mahmudova and Jabrailova [27], the main advantage of pairwise comparison was the ability to consider the “human factor” during decision-making. Some experts even combined pairwise comparison with fuzzy sets to tackle uncertainty and subjectivity judgments, towards expressing the factor importance over each other [28–30]. Therefore, the pairwise comparison was achieved for each factor and subfactor, leading to the identification of high- and low-priority factor distributions.

### 4.3. Weighting and Priority

In this study, the factor weights were determined to clarify the importance level. This indicated the priority level on each factor, based on the measurement data and information, as well as the reflection of different degrees. The results showed that the weight value was between 0 and 1, with the sum being observed at 1. This indicated that the higher weight value led to greater priority and importance level. In this study, the weighting was evaluated on each factor, subfactor, and combination.

#### 4.3.1. Factor Weighting

**Figure 8:**
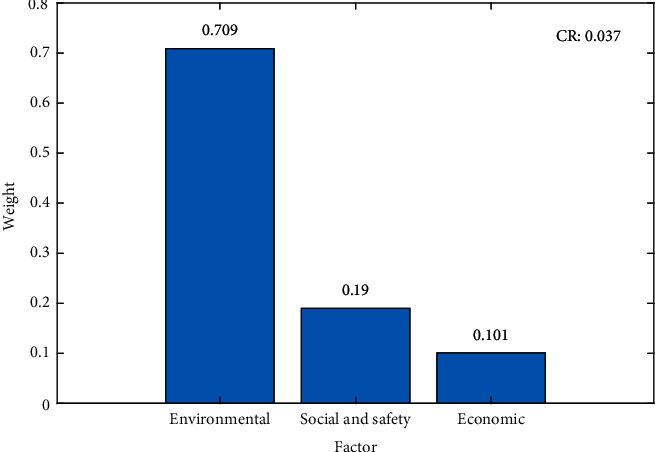
The weights on each factor using AHP.

The weight of each factor was successfully generated by AHP analysis, where the results varied between 0.101 and 0.709, that is, 0.709, 0.19, and 0.101 for environmental, social and safety, as well as economic variables, respectively. The weight distribution for each factor is shown in Figure 8, where the environmental factor had the highest value compared to others. This indicated that the highest priority in determining landfills is the environmental factor. The results also showed that the next low-weight values were orderly observed in the social/safety and economic factors, subsequently indicating a less significant difference. This was in line with Alfonso-Cardero et al. [31], who stated that the environment was the most crucial factor with the highest weight (0.61), compared to others such as techno-economic (0.36) and social (0.27) factors. The MSW generation was found to increase daily, subsequently leading to environmental degradation and pollution [32], due to the rapid development of the world population, urbanization, high material consumption, product complexity, and substances. The solid waste management hierarchy also contained prevention, minimization, reuse, recycle, energy recovery, and disposal. Meanwhile, the mismanagement of municipal waste directly affected environmental degradation, as improper methods of segregation and disposal polluted the soil and water. In this study, all the experts agreed that the environment deserved the highest priority, for determining a suitable landfill site for energy generation. Meanwhile, several previous studies argued against environmental factors being crucial in determining a suitable location [31–35]. These arguments indicated that suitable site selections should not only focus on one factor, as other various aspects should also be considered during the decision-making process.

In this study, the factors determining landfills for energy generation were divided into three parts, namely, environmental, social, and economic variables [5]. These factors should be adequately considered, as inappropriate locations often led to various problems, especially in the environment. Moreover, the environmental, social, and economic factors differed from one region to another, depending on the local conditions and situations [36]. In site selection, the location should also meet the local regulations, where social studies are needed to instantly avoid public conflicts.

#### 4.3.2. Weighting the Subfactor Division

Each factor contained a weighted subfactor for all divisions, where the distributions are shown in Table 7. Firstly, various subfactors weighed between 0.056 and 0.300 in the environmental division. This was based on the AHP analysis, where the highest to lowest importance weights were the distances from the settlement, river, sensitive regions, agricultural area, land slope, and climate. The results indicated that the distance from the settlement was the highest priority in determining the landfills for energy generation, compared to other subfactors. Secondly, the public acceptability and distance from the electrical grid, respectively, occupied the highest and lowest priority positions in the social and safety division. Meanwhile, the distance from the major road had the highest rank in the economic division (0.47), compared to the local road length (0.247), existing land use (0.202), and field cost (0.080).

#### 4.3.3. Overall Weighting of Subfactors Combination

This study showed the weights on the overall combinations (Table 8), where all subfactors weighed between 0.015 and 0.151. The results showed that the highest and lowest weights were the river distance and land cost, respectively. This indicated that the river distance was the most important parameter in determining a suitable landfill location for energy generation. According to Hariz et al. [37], the landfill distance from the river should be carefully considered, to minimize the contamination risk of water bodies. Although the subfactors proposed in this study were site-specific in Jabodetabek, Feyzi et al. still showed similar results, that is, the river distance had the highest weight. In addition, an exciting point in this AHP analysis showed that the distance from major road and existing land use variables had similar ratings, that is, rank 11. This was because both factors had similar weight values.

### 4.4. Evaluation

The weights occupied by each factor and subfactor were assessed using the CR evaluation metric, which ensured that the results generated by the MCDA were consistent and accurate. These weights were subsequently utilized for various purposes, such as academic, reports, and policy. Based on numerical analysis, the overall CR values for the factor (Figure 8), subfactor (Table 7), and combination (Table 8) were less than 0.1. CR = 0 indicates that the justification was perfectly consistent. Therefore, the overall weights produced were consistent and accurate. Several previous studies also showed consistent values (CR < 0.1) in the landfill site selection within various areas, such as Konya [38], Dhaka [39] Ahvaz [40], and Asir [41] in Turkey, Bangladesh, Iran, and Saudi Arabia, respectively. According to Saaty [42], the CR was improved by maintaining a small group of factors, sustaining the homogeneity of the variables within each category and comprehensively understanding the problem.

## 5. Implication and Limitation

### 5.1. Implication

**Figure 9:**
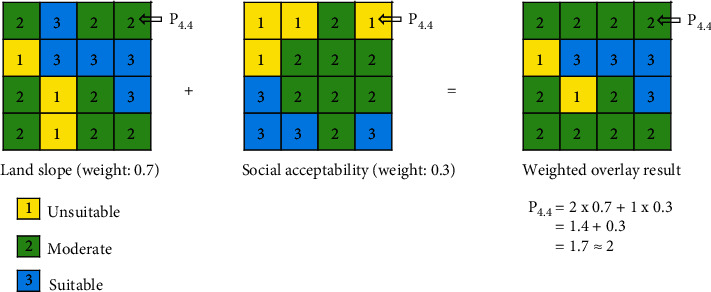
The weighted overlay analysis between two subfactors. Each coordinate in the pixel reflects the location. Parameters 1 (yellow), 2 (green), and 3 (blue) indicate the unsuitable, moderate, and suitable location classes for landfill site selection, respectively. The calculation step of the weighted overlay at each location is represented by pixel coordinate P_4,4_ (modified from Wijayanto et al. [45]).

This study showed the factors determining a suitable landfill location for MSW energy generation in Jabodetabek, Indonesia. In this study, the weight obtained was a valuable property, which should be used for weighted overlay analysis. This indicated that the weights of the various criteria were summed to calculate the total suitability [43]. Using the ArcGIS overlay tool, the resultant factor-based suitable site map was produced. This tool was found to manage large volumes of spatial data, as well as effectively and efficiently analyze data [6]. Several previous studies had also massively used the GIS tool, to analyze site suitability such as biodigester installation [44], broiler closed-house farm [45], landfill area [6], and solar fields [46]. A simple illustration of weighted overlay analysis is shown in Figure 9, where the results at each location occupying the pixel coordinate were calculated using equation (3), where *S*_*f*_*i*__ = the suitable class category at a certain location for the *i* factor and *w*_*f*_*i*__ = the weighted value of the *i* factor. These subfactors were subsequently overlaid to produce a factor-based final map, where the weights obtained played a critical role in future analysis. In addition, the weights on each factor were used to identify a suitable landfill site for energy generation, which is a direction for future studies.

(3) $$\begin{array}{c} P_{jk}=S_{f_{1}}w_{f_{1}}+S_{f_{2}}w_{f_{2}}+\cdots +S_{f_{i}}w_{f_{i}}. \end{array}$$

Landfills have reportedly become the third-largest source of anthropogenic methane emissions, after agriculture and fermentation [47]. This is due to releasing gases as a product of waste biodegradation, which primarily contains methane (CH_4_) and carbon dioxide (CO_2_). The CH_4_ generated in this phenomenon was captured and used as a renewable energy source [48]. Landfill gas is also a product of anaerobic decomposition, which occurs through physical, chemical, and microbial processes. Furthermore, the standard mathematical method used to estimate the CH_4_ content is the landfill gas emissions model (LandGEM) and intergovernmental panel on climate change (IPCC). In this study, the potential for converting waste into energy was vast in Jabodetabek. This was because the Jabodetabek metropolitan area potentially provided MSW energy of 820.90 GWh in 2020, based on the IPCC model [13]. In addition, this study is likely to serve as an input for the government as a policymaker. For example, this study showed that environmental factors were the highest priority in determining landfill location for energy generation. This indicates that the government should be more focused on completing the appropriate environmental criteria for landfill siting.

The AHP approach was applied to deal with the complexity of the various factors determining appropriate locations. Besides that, many approaches were also utilized, such as linear combination, ordered weighted average, analytical network process (ANP), fuzzy, TOPSIS (technique for order of preference by similarity to the ideal solution), and TODIM (Tomada de Decisão Interativa Multicritério). Each approach has several advantages and disadvantages capable of changing the final result, indicating that the right technique should be carefully selected by considering accuracy, convenience, complexity, user needs, and computation. Subsequently, these approaches were combined to produce fair decisions, such as AHP-fuzzy TOPSIS [35], fuzzy-AHP [40], and fuzzy-ANP [49]. It is also essential for an innovative selection method capable of efficiently reclaiming, evaluating, and displaying spatial results, based on the coordinates and attributes of each site. Also, a rigorous algorithm was previously proposed by Kyriakis et al. [50], to determine the location and waste-to-energy size. This was based on technical factors such as the amount of MSW available for incineration and the net calorific value.

### 5.2. Landfill Criteria for Energy Generation

MSW management is an essential environmental task, which includes various socioeconomic issues primarily concerned with the interplay between the social processes and economic activities within a society. In selecting a suitable landfill site, the socioeconomic factors to be considered include population density, distance from a major road, and land cost [35]. These factors play an essential role in evading continuous trouble and long-term effects on the environmental components such as groundwater contamination, surface fluid, and soil. Based on this study, socioeconomic factors were involved in the AHP analysis, to determine the appropriate landfill location. The complete factor should enable the policymakers to link unrelated sources of information, as well as analyze, visualize, and organize long-term planning objectives for better decision-making.

According to Table 9, the suitable criteria of various subfactors determining suitable landfill sites for energy generation are observed. This indicated that suitable criteria were imported from various sources and adapted by landfill sites for energy generation. In each subfactor, the display of values or justification was considered for the suitable criteria. Besides the established criteria, this led to low, moderate, and unsuitable groups. A complete description of each subfactor is shown in Table 9.

#### 5.2.1. Distance from Settlement

The distance between landfill and settlement should be carefully evaluated, as the landfill frequently has a negative impact on a variety of factors, including odors, noise, health issues, property values, and atheist points. Therefore, landfill location should not be very far or close to settlements and urban areas. Based on the literature, the appropriate landfill site to the settlement distance was >3,000 m.

#### 5.2.2. Distance from River

A landfill is a potential threat to rivers, wetlands, ponds, and lakes, due to the production of leachate and gaseous pollutants. Therefore, these locations should not be situated near any surface water. The landfill distance to the river should also be more than 2,500 m. According to Kamdar et al. [43], a distance of more than 900 m from the river was considered safe for a landfill site. However, Jabodetabek is a flood-prone area, indicating that the distance of >2,500 between the river and landfill is an appropriate value.

#### 5.2.3. Land Slope

The land slope is an essential factor for waste site selection, indicating that a steep terrain leads to higher excavation costs than flat surfaces. The suitable criteria for this factor are between 0 and 10%.

#### 5.2.4. Distance from Sensitive Areas

The Jabodetabek area contains several historical and sensitive areas, including museums, presidential palaces, waterfalls, temples, and national parks. According to Kamdar et al. [43], every ancient monument or the historical site was unsuitable for a landfill location. This indicates that the permissible distance between these areas and the landfill site should be more than 2,500 m.

#### 5.2.5. Distance from Agricultural Area

Landfill should not be located in an agricultural area, due to adversely affecting crop cultivation. Plants are also found to die through unsuitable conditions caused by the waste installation in the vicinity. This was in line with several studies, which stated that agricultural land was prohibited from being used as a landfill site [56]. Based on the literature, the distance of landfills to the agricultural area should be more than 300 m.

#### 5.2.6. Climate

Climatic conditions also need to be considered in selecting a suitable landfill site, due to rainfall being an essential factor in the climate subfactor. According to Baziene et al. [51], the pollution level in landfills decreased significantly when rainfall was low, and vice versa. This indicated that a suitable waste location should be situated in a low rainfall area.

#### 5.2.7. Social Acceptability

Although the consideration of a social factor is challenging based on suitable landfill selection, it should still importantly be identified. This indicates that acceptability expresses opinions regarding project realization in a suitable landfill location. Some areas are likely to also have different characteristics, depending on the original culture. Also, a landfill location should be selected based on the good ratings of social acceptability (no social conflict). This indicates that the community is willing to support and participate in establishing a landfill.

#### 5.2.8. Future Urban Growth

Future urban growth is an important parameter for determining landfills, due to the reduction in waste when the future population decreases in an area. This should be avoided because a small amount of debris is likely to lead to the inadequacy of converting energy from waste. Therefore, policymakers should import waste from other regions. This is because the appropriate future urban growth model in Jabodetabek is open space [39].

#### 5.2.9. Distance from Low Land

Low lands are not often higher than 200 m above sea level, where more water is held from rain or irrigation. When located very close to a low-lying area, the landfill is likely to encounter flooding. According to Akther et al. [39], most lowlands were used for various future purposes, including landfill sites. However, this should be adequately considered, as the suitable distance between low land and landfill is found to be >50 m.

#### 5.2.10. Distance from Electrical Grid

In Jabodetabek, electricity is entirely handled by a state company, that is, PT. PLN, where the electrical grid (as an energy supplier) is essential for energy generation installation. Therefore, the landfill should not be too far from the electrical grid. The suitable distance between this location and the grid should be <200 m.

#### 5.2.11. Distance from Major Road

The landfills often have a negative aesthetic impact on users, due to being near a major road. This indicates that the situation of this location close to the major road is likely to burden the budget, due to the high price of land. For easy access, landfills should not be very far from the main road. Therefore, this location should be within a reasonable distance from the major road. This was in line with Rezaeisabzevar et al. [5], which stated that the appropriate distance of the landfill from the major road was 1,000–2,000 m.

#### 5.2.12. Distance from Local Road

The Jabodetabek area has many interconnected local roads, with some so unpassable by garbage trucks due to being narrow. Therefore, the garbage trucks should have smooth access to pick up the pile. The distance from the landfill to the appropriate local road is <100 m.

#### 5.2.13. Existing Land Use

Land use describes the human utilization of available fields and the natural environment [43]. A land-use group contains forest, agricultural, residential, industrial, military, and archaeological areas. However, the residential, forests, and tourist areas are considered unsuitable for landfills. Based on Chabuk et al. [55], unused land was suitable for landfills.

#### 5.2.14. Land Cost

Most land ownership in Jabodetabek is dominated by the government, private owners, and real estate developers. This indicates that land prices are relatively high in this area, compared to other sites. Therefore, the low-cost lands suitable for technical and nontechnical landfill criteria are in great demand. According to Akther et al. [39], land types correlated with assets and were divided into six groups, namely, restricted (commercial offices), residential, park, public facility, mixed areas, and open land. Based on the analysis, open land had the suitable potential for landfill site placement.

### 5.3. Strengths, Weaknesses, Opportunities, and Threats (SWOT) Analysis

A total of 14 subfactors were subsequently grouped and analyzed using a SWOT approach. According to Ekmekçioglu et al. [57], the SWOT perspective applied two criteria through the simultaneous consideration of the internal and external environments, to obtain a systematic approach and support decision situations. The internal and external criteria were also selected with controllable and uncontrollable factors during decision-making. Furthermore, strengths and weaknesses as well as opportunities and threats were grouped into the internal and external environments, respectively. In this study, the grouping of subfactors into internal or external criteria is shown in Table 10. There were also 8 and 6 subfactors categorized as internal and external criteria, respectively. Based on this study, a more comprehensive SWOT analysis categorized each subfactor into groups of strengths, weaknesses, opportunities, or threats, to provide a systematic approach to decision-making. This focused on transforming weaknesses and threats into strengths and opportunities, respectively, which is an interesting direction for future studies. Also, several previous studies were found to combine AHP with SWOT for specific purposes, such as nuclear plant site selection [57], solar power project [58], sustainable manufacturing strategy [59], and strategic renewable energy resources selection [60].

### 5.4. Limitation of the Study

This study described various factors determining the landfill site, especially the installation of technology at a specific location, to convert waste into energy. However, several limitations should be highlighted towards future improvements, for the outputs to be adequately utilized by various parties:

- This report only used the Jabodetabek area as a case study, subsequently limiting other experts from adopting the weights. Therefore, cultural and site-specific characteristics with different environmental, social, and economic conditions should be considered before applying weights for further analysis.
- The weights normalization process in each factor should be carried out when some factors are excluded in a specific case due to insufficient data.
- This study is focused on determining the factors suitable for landfill site selection while considering the location suitability for energy generation as the ultimate goal.

## 6. Conclusion

The landfill site selection for energy generation was a complex task requiring the consideration of various factors. This indicated that the MCDA approach performed adequately towards the production of acceptable results, subsequently providing accurate decisions. Using the MCDA results, priority was ranked on each factor in determining landfill location. These should be accessible to the policymakers and councils in the Jabodetabek metropolitan area. Based on this study, 3 factors and 14 subfactors were structured in the MCDA hierarchy, with the involvement of 10 experts to create a pairwise comparison, whose weight identified the priority level for each variable. This indicated that a higher weight value led to greater factor priority. According to numerical analysis, the overall weight stored on the factors and subfactors was considered accurate and consistent, as confirmed by CR < 0.1. Therefore, this study confirmed that the environment was the highest-priority factor in determining a landfill site. This indicated that various parties, especially the government, should be more focused on completing appropriate environmental criteria for landfill site selection. The stored factor weights were also beneficial in determining the proper landfill location within Jabodetabek, through weighted overlay analysis. This should be investigated further as a future study direction.

## Figures and Tables

**Table 1:** The factors and subfactors symbol.

Factors	Subfactors	Symbol
Environmental (*V*)	Distance from settlement	*V*_1_
	Distance from river	*V*_2_
	Land slope	*V*_3_
	Distance from sensitive areas	*V*_4_
	Distance from the agricultural area	*V*_5_
	Climate	*V*_6_

Social and safety (*S*)	Social acceptability	*S*_1_
	Future urban growth	*S*_2_
	Distance from low land	*S*_3_
	Distance from the electrical grid	*S*_4_

Economic (*E*)	Distance from major road	*E*_1_
	Distance from local road	*E*_2_
	Existing land use	*E*_3_
	Land cost	*E*_4_

**Table 2:** Pairwise comparison on each factor.

	*V*	*S*	*E*
*V*	1.00	4.60	5.70
*S*	0.22	1.00	2.31
*E*	0.18	0.43	1.00

**Table 3:** Pairwise comparison on environmental subfactor.

	*V*_1_	*V*_2_	*V*_3_	*V*_4_	*V*_5_	*V*_6_
*V*_1_	1.00	2.45	3.25	1.53	1.72	2.83
*V*_2_	0.41	1.00	3.73	2.79	2.92	3.40
*V*_3_	0.31	0.27	1.00	1.23	1.57	2.12
*V*_4_	0.65	0.36	0.82	1.00	1.43	3.80
*V*_5_	0.58	0.34	0.64	0.70	1.00	3.80
*V*_6_	0.35	0.29	0.47	0.26	0.26	1.00

**Table 4:** Pairwise comparison on social and safety subfactor.

	*S*_1_	*S*_2_	*S*_3_	*S*_4_
*S*_1_	1.00	3.72	3.40	3.33
*S*_2_	0.27	1.00	1.19	2.19
*S*_3_	0.29	0.84	1.00	1.63
*S*_4_	0.30	0.46	0.61	1.00

**Table 5:** Pairwise comparison on economic subfactor.

	*E*_1_	*E*_2_	*E*_3_	*E*_4_
*E*_1_	1.00	3.27	2.31	3.30
*E*_2_	0.55	1.00	1.83	3.63
*E*_3_	0.28	0.55	1.00	3.98
*E*_4_	0.00	0.28	0.21	1.00

**Table 6:** Pairwise comparison on subfactor combination.

	*V*_1_	*V*_2_	*V*_3_	*V*_4_	*V*_5_	*V*_6_	*S*_1_	*S*_2_	*S*_3_	*S*_4_	*E*_1_	*E*_2_	*E*_3_	*E*_4_
*V*_1_	1.00	2.45	3.25	1.53	1.72	2.83	1.76	2.23	2.73	3.52	3.43	3.33	2.84	4.51
*V*_2_	0.41	1.00	3.73	2.79	2.92	3.40	2.58	2.80	2.33	3.52	4.25	4.07	2.17	5.31
*V*_3_	0.31	0.27	1.00	1.23	1.57	2.12	2.28	3.25	2.04	3.31	3.83	3.47	2.14	3.75
*V*_4_	0.65	0.36	0.82	1.00	1.43	3.80	1.83	2.92	2.63	4.00	4.60	4.32	2.38	5.32
*V*_5_	0.58	0.34	0.64	0.70	1.00	3.80	2.05	3.40	2.40	2.65	3.12	3.53	2.18	3.83
*V*_6_	0.35	0.29	0.47	0.26	0.26	1.00	1.90	2.79	2.19	2.17	2.45	2.52	2.64	3.74
*S*_1_	0.57	0.39	0.44	0.55	0.49	0.53	1.00	3.72	3.40	3.33	3.93	4.20	4.11	4.92
*S*_2_	0.45	0.36	0.31	0.34	0.29	0.36	0.27	1.00	1.19	2.19	2.77	2.98	3.25	4.23
*S*_3_	0.37	0.43	0.49	0.38	0.42	0.46	0.29	0.84	1.00	1.63	3.04	3.73	2.59	3.97
*S*_4_	0.28	0.28	0.30	0.25	0.38	0.46	0.30	0.46	0.61	1.00	3.73	3.80	2.37	5.32
*E*_1_	0.29	0.24	0.26	0.22	0.32	0.41	0.25	0.36	0.33	0.27	1.00	3.27	2.31	3.30
*E*_2_	0.30	0.25	0.29	0.23	0.28	0.40	0.24	0.34	0.27	0.26	0.55	1.00	1.83	3.63
*E*_3_	0.35	0.46	0.47	0.42	0.46	0.38	0.24	0.31	0.39	0.42	0.28	0.55	1.00	4.87
*E*_4_	0.22	0.19	0.27	0.19	0.26	0.27	0.20	0.24	0.25	0.19	0.00	0.28	0.21	1.00

**Table 7:** The weights distribution on each subfactor division.

Factors	Subfactors	Weight	Rank
Environmental CR: 0.097	Distance from settlement	0.300	1
	Distance from river	0.270	2
	Land slope	0.116	5
	Distance from sensitive areas	0.140	3
	Distance from the agricultural area	0.118	4
	Climate	0.056	6

Social and safety CR: 0.031	Social acceptability	0.518	1
	Future urban growth	0.222	2
	Distance from low land	0.147	3
	Distance from the electrical grid	0.113	4

Economic CR: 0.097	Distance from major road	0.471	1
	Distance from local road	0.247	2
	Existing land use	0.202	3
	Land cost	0.080	4

**Table 8:** The weight on the overall subfactors (CR = 0).

Subfactors	Weight	Rank
Distance from settlement	0.143	2
Distance from river	0.151	1
Land slope	0.098	4
Distance from sensitive areas	0.111	3
Distance from the agricultural area	0.096	5
Climate	0.067	7
Social acceptability	0.086	6
Future urban growth	0.050	9
Distance from low land	0.051	8
Distance from the electrical grid	0.045	10
Distance from major road	0.031	11
Distance from local road	0.025	12
Existing land use	0.031	11
Land cost	0.015	13

**Table 9:** The suitable criteria of landfill for energy generation.

Goal	Factors	Subfactors	Suitable criteria	Reference(s)
Landfill for energy generation from MSW	Environmental	Distance from the settlement (m)	>3,000	[6]
		Distance from the river (m)	>2,500	[39]
		Land slope (%)	0–10	[38]
		Distance from sensitive areas (m)	>2,500	[43]
		Distance from the agricultural area (m)	>300	[40]
		Climate	Low rainfall	[51]
	Social and safety	Social acceptability	Good	[7, 52]
		Future urban growth	Open space	[39]
		Distance from low land (m)	>50	[39]
		Distance from electrical grid (m)	<200	[53]
	Economic	Distance from a major road (m)	1,000–2,000	[5]
		Distance from the local road (m)	<100	[54]
		Existing land use	Unused lands	[55]
		Land cost	Open land	[39]

**Table 10:** The classification of subfactors into internal or external criteria based on SWOT analysis.

No.	Criteria	Subfactor
1	Internal	Distance from low land
		Distance from the electrical grid
		Distance from major road
		Distance from local road
		Distance from settlement
		Distance from river
		Distance from sensitive areas
		Distance from the agricultural area

2	External	Climate
		Social acceptability
		Future urban growth
		Land cost
		Land slope
		Existing land use

## Data Availability

All the data supporting the results are included in the article.
